# Dexamethasone Protects Against Tourniquet-Induced Acute Ischemia-Reperfusion Injury in Mouse Hindlimb

**DOI:** 10.3389/fphys.2018.00244

**Published:** 2018-03-20

**Authors:** Ryan M. Corrick, Huiyin Tu, Dongze Zhang, Aaron N. Barksdale, Robert L. Muelleman, Michael C. Wadman, Yu-Long Li

**Affiliations:** Department of Emergency Medicine, University of Nebraska Medical Center, Omaha, NE, United States

**Keywords:** tourniquet, acute ischemia-reperfusion injury, skeletal muscle, neuromuscular junction, cytokine, dexamethasone

## Abstract

Extremity injuries with hemorrhage have been a significant cause of death in civilian medicine and on the battlefield. The use of a tourniquet as an intervention is necessary for treatment to an injured limb; however, the tourniquet and subsequent release results in serious acute ischemia-reperfusion (IR) injury in the skeletal muscle and neuromuscular junction (NMJ). Much evidence demonstrates that inflammation is an important factor to cause acute IR injury. To find effective therapeutic interventions for tourniquet-induced acute IR injuries, our current study investigated effect of dexamethasone, an anti-inflammatory drug, on tourniquet-induced acute IR injury in mouse hindlimb. In C57/BL6 mice, a tourniquet was placed on unilateral hindlimb (left hindlimb) at the hip joint for 3 h, and then released for 24 h to induce IR. Three hours of tourniquet and 24 h of release (24-h IR) caused gastrocnemius muscle injuries including rupture of the muscle sarcolemma and necrosis (42.8 ± 2.3% for infarct size of the gastrocnemius muscle). In the NMJ, motor nerve terminals disappeared, and endplate potentials were undetectable in 24-h IR mice. There was no gastrocnemius muscle contraction in 24-h IR mice. Western blot data showed that inflammatory cytokines (TNFα and IL-1β) were increased in the gastrocnemius muscle after 24-h IR. Treatment with dexamethasone at the beginning of reperfusion (1 mg/kg, i.p.) significantly inhibited expression of TNFα and IL-1β, reduced rupture of the muscle sarcolemma and infarct size (24.8 ± 2.0%), and improved direct muscle stimulation-induced gastrocnemius muscle contraction in 24-h IR mice. However, this anti-inflammatory drug did not improve NMJ morphology and function, and sciatic nerve-stimulated skeletal muscle contraction in 24-h IR mice. The data suggest that one-time treatment with dexamethasone at the beginning of reperfusion only reduced structural and functional impairments of the skeletal muscle but not the NMJ through inhibiting inflammatory cytokines.

## Introduction

Based on the data from the Center for Disease Control and Prevention, over 14 million persons with extremity injuries visit emergency rooms, with an estimated cost of $80 billion each year (Center for Disease Control and Prevention, 2017). Exsanguinating injury of the extremities is a key factor of trauma fatalities in civilian medicine and on the battlefield (Mabry, 2006; Inaba et al., 2015; Ode et al., 2015). Tourniquet use is a primary tool in first-line treatment of severe limb hemorrhage (Mabry, 2006; Beekley et al., 2008; Kragh et al., 2012; Welling et al., 2012). The tourniquet is also used to create an optimal operating fields with bloodless in vascular surgeries and orthopedic, musculoskeletal reconstructive procedures (an estimated 15,000 procedures daily in the US and elsewhere) (Tai et al., 2011; Halladin et al., 2014). However, acute ischemia-reperfusion (IR) injuries induced by a tourniquet use with subsequent reperfusion usually occur, which include tissue apoptosis and necrosis in the skeletal muscle, and severe structural and functional damage in the neuromuscular junction (NMJ) (Aho et al., 1983; Mohler et al., 1999; Schoen et al., 2007; Tran et al., 2011, 2012; Gillani et al., 2012). These acute injuries also affect long-term recovery of NMJ function and skeletal muscle contraction from IR injuries, even resulting in complete limb paralysis as well as the need for secondary amputation (Kam et al., 2001; Noordin et al., 2009; Vignaud et al., 2010). These complications have led to the de-emphasis and limitations of tourniquet use (Clasper et al., 2009; Kue et al., 2015).

Until now the cellular and molecular mechanisms responsible limb acute IR injuries are unclear. Some studies demonstrated that overproduction of inflammatory cytokines and reactive oxygen species in injured skeletal muscles during the acute IR causes neuronal damage and deficit of skeletal muscle function (Seekamp et al., 1993; Clementsen and Reikeras, 2008; Chen and Nunez, 2010; Eltzschig and Eckle, 2011). Based on this fact, inhibition of production and release of inflammatory cytokines is likely considered as one of the strategies for limb acute IR injury. Dexamethasone (Dex), a synthetic glucocorticoid and potent anti-inflammatory drug, is used clinically to treat many inflammatory and autoimmune diseases, including asthma, rheumatoid arthritis, lupus nephritis, inflammatory shock, etc. (Barnes, 2011). Therefore, we hypothesized that Dex could protect the limb from acute IR injury through inhibiting inflammatory cytokines because glucocorticoids reduce production and release of inflammatory cytokines in whole blood (Schwiebert et al., 1996; Smits et al., 1998; Schuld et al., 2001).

In general, both innervated motor nerve-muscle transmission (i.e., NMJ) and skeletal muscle are involved in skeletal muscle contractile function (Chervu et al., 1989; Fish et al., 1989; Eastlack et al., 2004). In this study, therefore, three purposes were performed: (1) to assess the morphological and functional changes of the skeletal muscle and NMJ; (2) to test whether Dex could attenuate the structural and functional impairment of the skeletal muscle and NMJ; (3) to measure the inhibitory effect of Dex on inflammatory cytokines in a murine model of tourniquet-induced 3-h ischemia and subsequent 24-h reperfusion.

## Materials and methods

### Animals

Totally 63 male C57/BL6 mice (21 sham, 21 tourniquet-induced IR, and 21 tourniquet-induced IR + Dex mice) weighing 22–25 g (Charles River Laboratory, Wilmington, MA) were used in this study. The mice were housed under controlled temperature and humidity and a 12:12-h dark–light cycle, and were provided water and mouse chow *ad libitum*. All experimental procedures were approved by the University of Nebraska Medical Center Institutional Animal Care and Use Committee and were carried out in accordance with the National Institutes of Health (NIH Publication No. 85–23, revised 1996) and the American Physiological Society's “Guides for the Care and Use of Laboratory Animals.”

### A mouse model of tourniquet-induced hindlimb IR and drug treatment

Tourniquet-induced mouse hindlimb IR is a mature animal model and has been used in our previous studies (Tran et al., 2011, 2012; Tu et al., 2017; Zhang et al., 2017). Briefly, under anesthesia with a cocktail consisting of 100 mg/kg ketamine and 10 mg/kg xylazine, given as an intraperitoneal injection (10 ml/kg body weight), the tourniquet in unilateral hindlimb (left hindlimb) was done for 3 h by setting an orthodontic rubber band at the hip joint, using a McGivney hemorrhoidal ligatorer. Then the orthodontic rubber band was released to begin reperfusion for 24 h. During the period of this procedure, the level of anesthesia was continuously monitored by testing the respiratory patterns and toe pinch reflex. A heating pad was used to maintain body temperature at 37°C until the animals woke up. Our previous study has verified skeletal muscle IR in this animal model through testing blood flow in IR gastrocnemius muscles (Tran et al., 2011). Before waking up, mice were kept well hydrated with an intraperitoneal injection of 0.2 ml normal saline every 2 h.

Usually the contralateral skeletal muscle is used as an uninjured control muscle in a unilateral tourniquet-induced IR model (Woitaske and McCarter, 1998; Walters et al., 2008; Lotfi et al., 2013). However, it is possible that metabolites from injured skeletal muscles and drug treatments are also distributed into contralateral NMJs and skeletal muscles, affecting their functions. A sham group of mice was designed as the control group in the present study to avoid this possibility. The same procedure except for the application of the orthodontic rubber band was performed in sham-operated animals.

Dex (Sigma-Aldrich, St. Louis, MO) was dissolved in 0.9% NaCl. IR mice were randomly divided into two groups for administration of normal saline or Dex (1 mg/kg), respectively. Normal saline solution or Dex (0.2 ml) was intraperitoneally administered at the beginning of reperfusion. All terminal experiments were performed on mice after 24 h of reperfusion.

### *In-situ* detection of the gastrocnemius muscle contractile force

The gastrocnemius muscle contractile force was measured in five sham, five tourniquet-induced IR, and five tourniquet-induced IR + Dex mice as described previously (Tu et al., 2017; Zhang et al., 2017). Briefly, under anesthesia (800 mg kg^−1^ urethane and 40 mg kg^−1^ chloralose, a mixing solution of 20% urethane and 1% chloralose, 4 ml kg^−1^, i.p.), the mouse was kept in prone position and maintained at 37°C. The isolated middle and distal ends of the left gastrocnemius muscle were moistened by warmed saline. The distal end of the gastrocnemius muscle was sutured and connected to a wide range force transducer (AD Instruments, Colorado Springs, CO). The left sciatic nerve was exposed through splitting the left biceps femoris muscle along muscle fibers. Maximal electrical tetanus stimulation (10 V, 50 Hz, 0.1-ms pulse, 5-s duration) was produced by a bipolar platinum electrode placed on the distal cut end of the sciatic nerve to induce tetanic contractile force of the gastrocnemius muscle. A similar procedure was performed for direct gastrocnemius muscle stimulation (10–50 V, 50 Hz, 0.1-ms pulse, 5-s duration). Considering spatial variability for direct gastrocnemius muscle stimulation, we selected 4 areas of gastrocnemius muscles for this measurement and collected these results as the data for one measured muscle. PowerLab 8/30 Data Acquisition Systems with LabChart 7 (AD Instruments, Colorado Springs, CO, USA) were used to record and analyze the muscle contractile force.

### Measurement of infarct size in the gastrocnemius muscle (Tran et al., 2011)

Infarct size of the gastrocnemius muscle induced by tourniquet-related IR was tested by triphenyl tetrazolium chloride assay. After measurement of the gastrocnemius muscle contractile force, mouse gastrocnemius muscle was quickly isolated and cut into 1.5 mm transverse slices. The cut slices were cleaned by cold saline solution, and subsequently incubated with 1% triphenyl tetrazolium chloride solution (Sigma-Aldrich, St. Louis, MO) at room temperature for 1 h. Slice images were digitalized by a Canon camera. In the obtained muscle images, the muscle with a dark purple red color stain was defined as the viable muscle, and the muscle with a pale brown color stain was defined as the infarcted muscle. After the viable muscle and infarcted muscle were quantified by Adobe Photoshop CS5, the ratio (%, wet weight/wet weight) of the infarcted muscle to total gastrocnemius muscle (viable plus infarcted muscles) was calculated as the infarct size.

### Histological evaluation of skeletal muscle damage and inflammatory infiltration

In the first experiment, gastrocnemius muscles from three sham, three tourniquet-induced IR, and three tourniquet-induced IR + Dex mice were successively fixed in Methacarn solution (300 ml methanol, 150 ml chloroform, and 50 ml acetic acid) for 48 h, and 60% ethanol for 72 h. The fixed muscle was embedded into paraffin wax after routine processing, and then cut into 4-μm-thick longitudinal sections. After deparaffinization, sections were stained with hematoxylin and eosin (H&E, Sigma-Aldrich, St. Louis, MO) for inflammatory infiltration and with Masson's trichrome (Sigma-Aldrich, St. Louis, MO) for skeletal muscle damage. Stained sections were captured by a bright-field microscope (Leica Microsystems Inc., Buffalo Grove, IL) with digital camera (Qimaging MicroPublisher 3.3RTV).

In the second experiment, gastrocnemius muscles from three sham, three tourniquet-induced IR, and three tourniquet-induced IR + Dex mice were postfixed with 4% paraformaldehyde for 12 h, and then soaked in 30% sucrose for 12 h at 4°C for cryostat procedure. The muscle was cut into 10-μm-thick cross-sections in a freezing cryostat at −20°C. Tissue sections were processed with standard immunocytochemical staining procedure. Briefly, tissue sections were permeabilized with 0.3% Triton X-100 (Thermo Fisher Scientific, Waltham, MA) in PBS at room temperature for 20 min. After tissue sections were successively incubated with 10% normal goat serum (Jackson Immunoresearch Labs Inc.), rabbit anti-laminin antibody (Sigma-Aldrich, St. Louis, MO), and goat anti-rabbit IgG labeled with Alexa Fluor 594 (Thermo Fisher Scientific, Waltham, MA), tissue images were obtained using a Leica fluorescent microscope with digital camera (Qimaging Retiga Exi Fast 1394).

### Electrophysiological recording of the EPP *in-situ*

The EPP recording was performed in five sham, five tourniquet-induced IR, and five tourniquet-induced IR + Dex mice as described previously (Tu et al., 2017; Zhang et al., 2017). Under anesthesia (800 mg/kg urethane and 40 mg/kg chloralose, i.p.), the isolated middle and distal end of the left gastrocnemius muscle was moistened by warmed saline solution. To avoid the influence of muscle contraction on the EPP recording, a specific muscle Na^+^ channel blocker, μ-conotoxin GIIIB (4 μM, 200 μL) was locally provided into the gastrocnemius muscle to inhibit muscle contraction. A glass microelectrode filled with 3 M KCl (5–15 MΩ pipette resistance) was slowly inserted into the gastrocnemius muscle fiber and connected with an intracellular preamplifier (IX1; Dagan Corporation, Minneapolis, MN, USA) for the EPP recording. When miniature endplate potentials (mEPPs) could be recorded to determine an endplate close to the electrode, the EPPs were recorded by intracellular recording technique under the electrical stimulation of exposed sciatic nerve (10 V, 50 Hz, 0.1 ms). Finally sciatic nerve stimulation-evoked EPPs was digitized by PowerLab 8/30 Data Acquisition System with LabChart 7 (AD Instruments), and stored on computer for analyzing the amplitude of EPPs. In the gastrocnemius muscle from each mouse, 6–8 sites were selected for EPPs recording.

### Immunohistochemistry of the NMJs (Tu et al., 2017)

After recording of the EPP in-situ, mouse gastrocnemius muscle was quickly isolated for postfixing with 4% paraformaldehyde for 15 min, and subsequently incubating with 0.1 M glycine for 15 min. To facilitate probe penetrations into NMJs, The gastrocnemius muscle was divided into 8–10 small longitudinal segments, and then permeabilized in −20°C methanol for 10 min. After blocking with PBS containing 0.5% Triton and 1% BSA for 1 h, small longitudinal segments of the gastrocnemius muscle were incubated overnight at 4°C in a cocktail of primary antibodies, including mouse anti-neurofilament 200 (Sigma-Aldrich, St. Louis, MO) and rabbit anti-synaptophysin (Thermo Fisher Scientific, Waltham, MA) antibodies for axon and nerve terminal labeling. Then muscle segments were incubated overnight at 4°C with Alexa Fluor® 594 labeled donkey anti-mouse (Thermo Fisher Scientific, Waltham, MA) and anti-rabbit (Thermo Fisher Scientific, Waltham, MA) IgGs, and Alexa Fluor® 488 labeled α-Bungarotoxin (α-BTX, Thermo Fisher Scientific, Waltham, MA). Finally, images of muscle segments mounted on glass slides were captured using a Leica fluorescent microscope (Leica DMR, Leica Microsystems Inc., Buffalo Grove, IL) to analyze immunohistochemically labeled NMJs including motor nerve terminals and nAChR clusters.

In each muscle segment, five different regions were selected to obtain Z-stack images of the NMJ. All analyses were done by ImageJ software (NIH Image) on en-face NMJs. In the NMJ, the percentage of motor nerve innervation to nAChR clusters was quantified by measurements of nerve terminal sizes labeled with neurofilament and synaptophysin. The endplate with or without labeling for neurofilament and synaptophysin was defined as an innervated or denervated endplate, respectively. The nAChR areas in NMJs labeled with α-BTX were used to calculate the area per fragment, the nAChR area per nAChR cluster, and the number of discrete fragments per nAChR cluster. A fragmented nAChR cluster was defined when the number of discrete fragments per nAChR cluster ≥5.

### Protein expression of Il-1β and TNFα in gastrocnemius muscles

Gastrocnemius muscles from five sham, five tourniquet-induced IR, and five tourniquet-induced IR + Dex mice were rapidly removed and stored at −80°C until analyzed. A lysing buffer (10 mM Tris, 1 mM EDTA,150 mM NaCl, 1% SDS, 1 mM PMSF; pH 7.4) plus protease inhibitor cocktail (100 μl/ml, P2714, Sigma-Aldrich, St. Louis, MO) was added into the muscle tissue for homogenization. Homogenized muscle tissue was centrifuged at 12,000 *g* for 20 min at 4°C. Total protein concentration in the supernatant was measured by a bicinchoninic acid protein assay kit (Thermo Fisher Scientific, Waltham, MA). Same volume of the loading buffer was added into protein samples to mix and heat for 5 min at 100°C. Protein samples (40 μg/well) loaded in the stacking gel were separated on a 12% sodium dodecyl sulfate (SDS)-polyacrylamide running gel. Proteins of these samples were electrophoretically transferred at 200 mA for 3 h onto PVDF membrane (EMD Millipore, Billerica, MA). The membrane was blocked with 5% non-fat milk for 1 h. Then the membrane was probed with rabbit anti TNF-α antibody (Cell Signaling Technology, Beverly, MA), mouse anti IL-1β antibody (Cell Signaling Technology, Beverly, MA), or mouse anti β-actin antibody (Santa Cruz Biotechnology, Dallas, TX) overnight at 4°C. After washing by PBS, the membrane was incubated with peroxidase-conjugated goat anti-rabbit IgG (Thermo Fisher Scientific, Waltham, MA), or goat anti-mouse IgG (Thermo Fisher Scientific, Waltham, MA) for 1 h. The signal was developed by enhanced chemiluminescence substrate (Thermo Fisher Scientific, Waltham, MA), and captured by UVP bioimaging system (UVP, Upland, CA).

### Statistical analysis

All data are presented as means ± SE. SigmaPlot 12 was used for data analysis. A one-way ANOVA with *post hoc* Bonferroni test was used to determine statistical significance for multi group comparison. A Chi-Square test was performed for percentage of nerve innervation in the NMJ. Normal distribution of data was confirmed with Kolmogorov–Smirov test and equal variance with Levene's test. Statistical significance was accepted when *p* < 0.05.

## Results

### Effects of dex on acute IR-induced morphological and functional changes in gastrocnemius muscles

**Figure 1:**
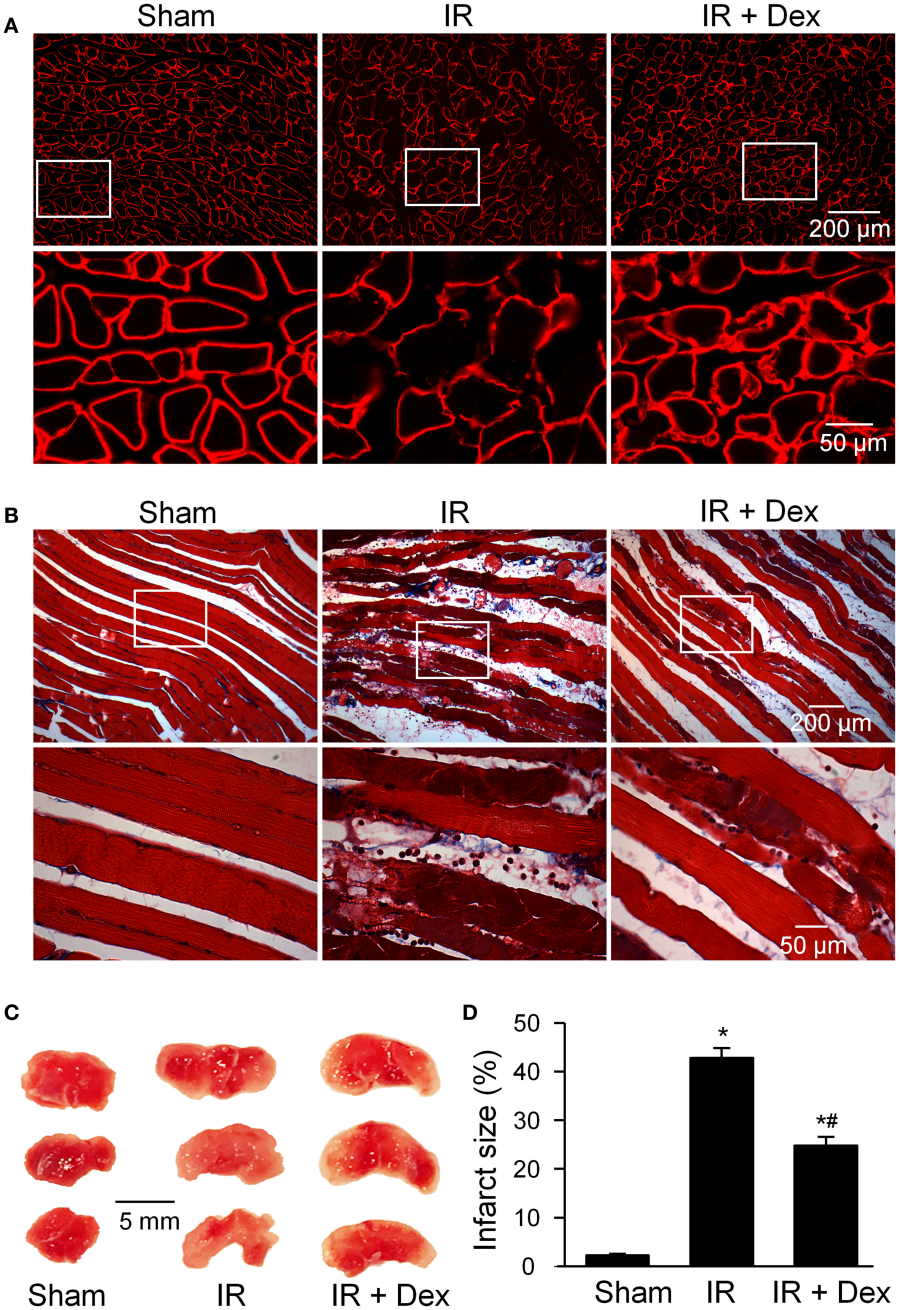
The morphology of gastrocnemius muscles in all experimental groups. **(A)**, histology in the cross section of gastrocnemius muscles, measured by laminin staining (a marker for muscle sarcolemma). **(B)**, histology in the longitudinal section of gastrocnemius muscles, measured by Masson's trichrome staining. **(C,D)**, representative and summary data for infarct size in gastrocnemius muscles. Data are mean ± SEM, *n* = 5 mice in each group. ^*^P < 0.05 vs. sham; ^#^*P* < 0.05 vs. IR.

Acute IR-induced gastrocnemius muscle injuries were shown in Figure 1. An obvious breakage of muscle fiber was observed in tourniquet-induced IR group of mice, revealed by laminin staining of gastrocnemius muscles in cross-section (Figure 1A). Tourniquet-induced IR caused undulating muscle cell borders, irregular spacing of muscle striations, and disruption of muscle fibers, evidenced by Massion's trichrome staining of gastrocnemius muscles in longitudinal-section (Figure 1B). Additionally, infarct size of the skeletal muscle was measured by triphenyl tetrazolium chloride assay. Tourniquet-induced IR also markedly resulted in infarction in gastrocnemius muscles (42.8 ± 2.4%), compared to that in sham group of mice (0%, *p* < 0.05, Figures 1C,D). Dex treatment (1 mg/kg, i.p.) at the beginning of reperfusion significantly attenuated, but did not abolish, acute IR-induced gastrocnemius muscle injuries including broken muscle sarcolemmata, disrupted muscle fibers, and muscle infarct size, measured by above three methods (Figure 1).

**Figure 2:**
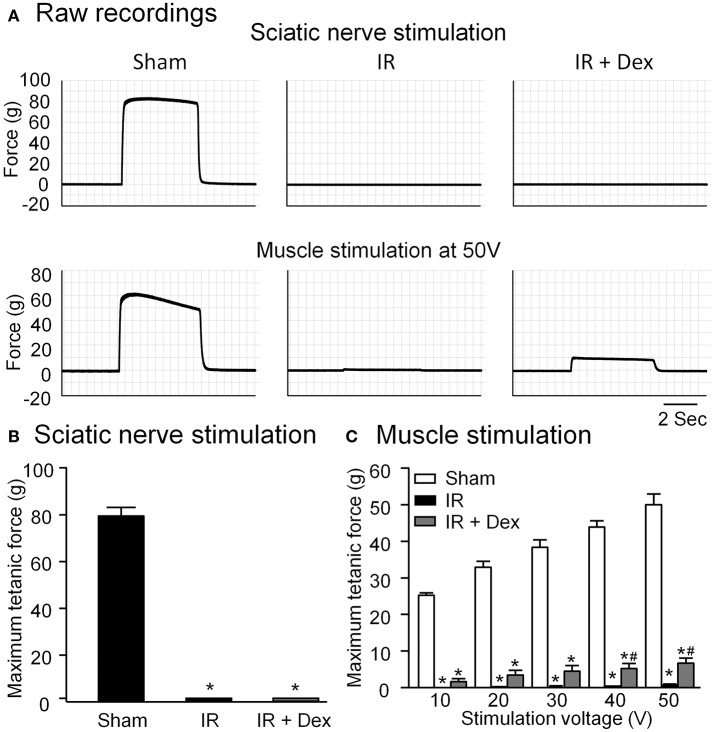
Representative **(A)** and summary **(B,C)** data for in situ gastrocnemius muscle tetanic contraction elicited by sciatic nerve stimulation or direct muscle stimulation in all experimental groups. Data are mean ± SEM, *n* = 5 mice in each group. ^*^P < 0.05 vs. sham; ^#^*P* < 0.05 vs. IR.

Gastrocnemius muscle contraction was measured by sciatic nerve stimulation or direct muscle stimulation, which can determine if IR-induced skeletal muscle contractile dysfunction is caused by muscle injury alone and/or by nerve-muscle transmission failure (Chervu et al., 1989; Eastlack et al., 2004). In tourniquet-induced IR group, both sciatic nerve stimulation and direct muscle stimulation did not evoke gastrocnemius muscle contraction (Figure 2). Dex treatment improved direct muscle stimulation-induced muscle contraction in IR-injured gastrocnemius muscles (*P* < 0.05 vs. IR group, Figure 2C), whereas it did not affect sciatic nerve stimulation-induced gastrocnemius muscle contraction (Figure 2B).

### Effects of dex on structural and functional alterations in NMJs

**Figure 3:**
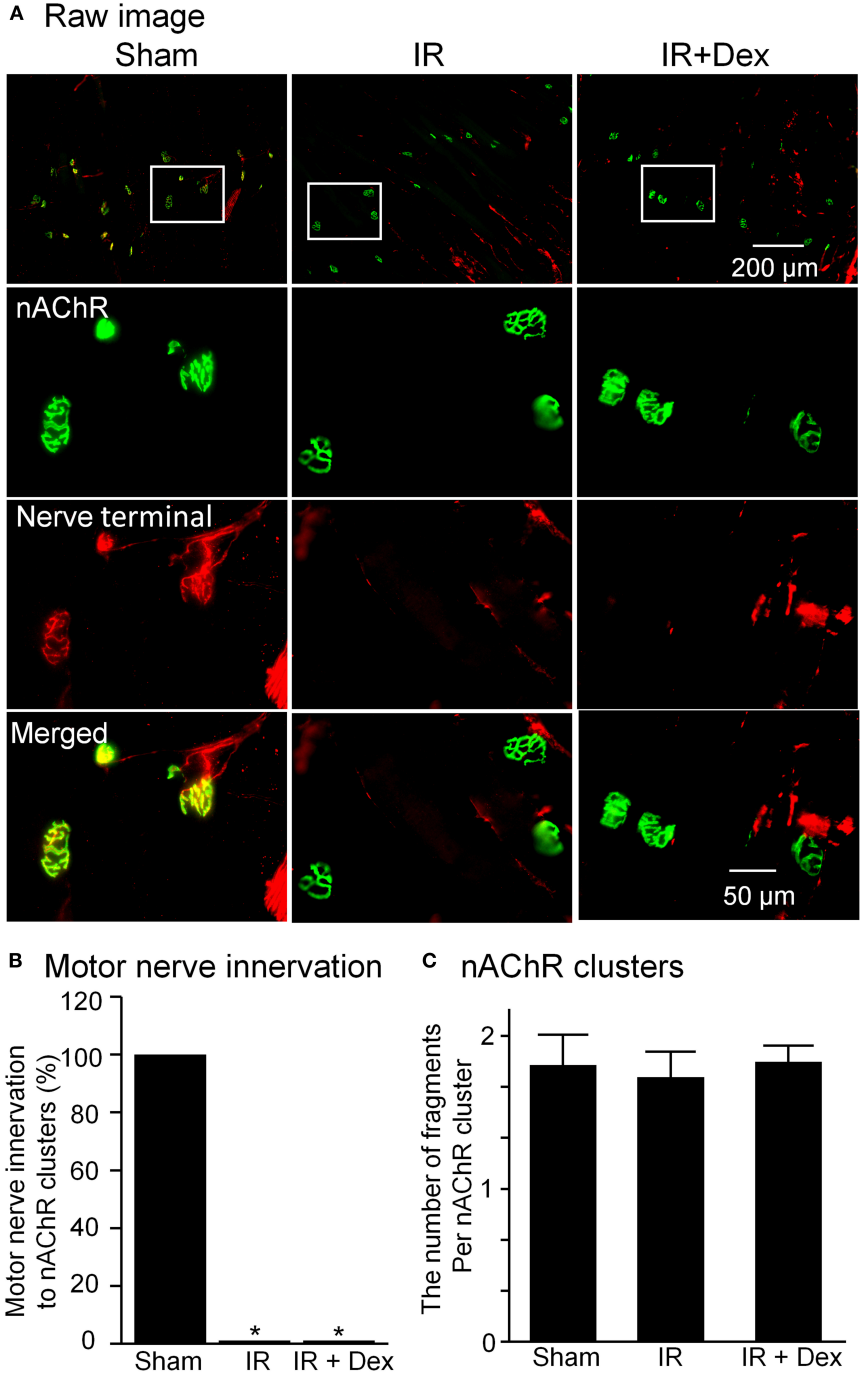
The morphology of NMJs including motor nerve terminals and nAChRs located on gastrocnemius muscles from all experimental groups. **(A)**, representative images of NMJs. Synaptophysin and neurofilament 200 (red color) and α-bungarotoxin (BTX, green color) were used to label motor nerve terminals and nAChR clusters in NMJs. **(B,C)**, mean data for motor nerve innervation to nAChR clusters and the number of fragments per nAChR cluster. Data are mean ± SEM, *n* = 5 mice in each group. ^*^P < 0.05 vs. sham.

In Figure 3, motor nerve terminals labeled by neurofilament and synaptophsin and nAChR clusters labeled by BTX were investigated. As a major feature of NMJs, a specific synapse is normally formed by the presynaptic membrane (motor nerve terminals) and postsynaptic membrane (nAChR clusters), which can transmit the signal from motor neurons to skeletal muscles (Figure 3A). In IR group, the gastrocnemius muscle was totally denervated by motor nerve terminals in all NMJs (0% motor nerve innervation to nAChR clusters, *p* < 0.05 vs. sham). Compared to sham mice, the structure of nAChR clusters did not show significant alterations, and the number of fragments per nAChR cluster had no markedly changes (1.65 ± 0.13 vs. sham 1.80 ± 0.21, Figure 3C). Treatment with Dex (1 mg/kg) did not blunt IR-induced disappearance of motor nerve terminals in NMJs from the gastrocnemius muscle (Figure 3).

**Figure 4:**
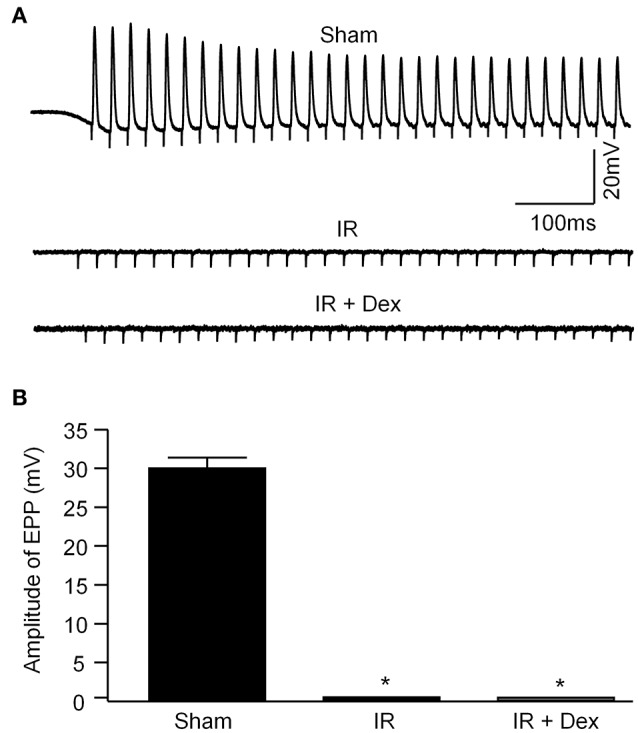
Representative **(A)** and summary **(B)** data for sciatic nerve-stimulated endplate potentials (EPPs) recorded in gastrocnemius muscles from all experimental groups. Data are mean ± SEM, *n* = 5 mice in each group. ^*^P < 0.05 vs. sham.

As noted above, electrical signals are usually transmitted from motor neurons to skeletal muscles through the NMJ (including motor nerve terminals and nAChR clusters), which can trigger skeletal muscle contraction. Recording of sciatic nerve-stimulated EPPs was used to NMJ function in the gastrocnemius muscle (Figure 4). In sham animals, sciatic nerve stimulation evokes regular, steady EPPs and the amplitude of EPPs was 29.9 ± 1.4 mV. In the IR group, sciatic nerve-stimulated EPPs were not detectable in all NMJs (Figure 4). When Dex (1 mg/kg) was administered at the beginning of reperfusion, IR-induced disappearance of sciatic nerve-stimulated EPPS was not improved (Figure 4).

### Effects of dex on inflammatory cytokines in IR gastrocnemius muscles

**Figure 5:**
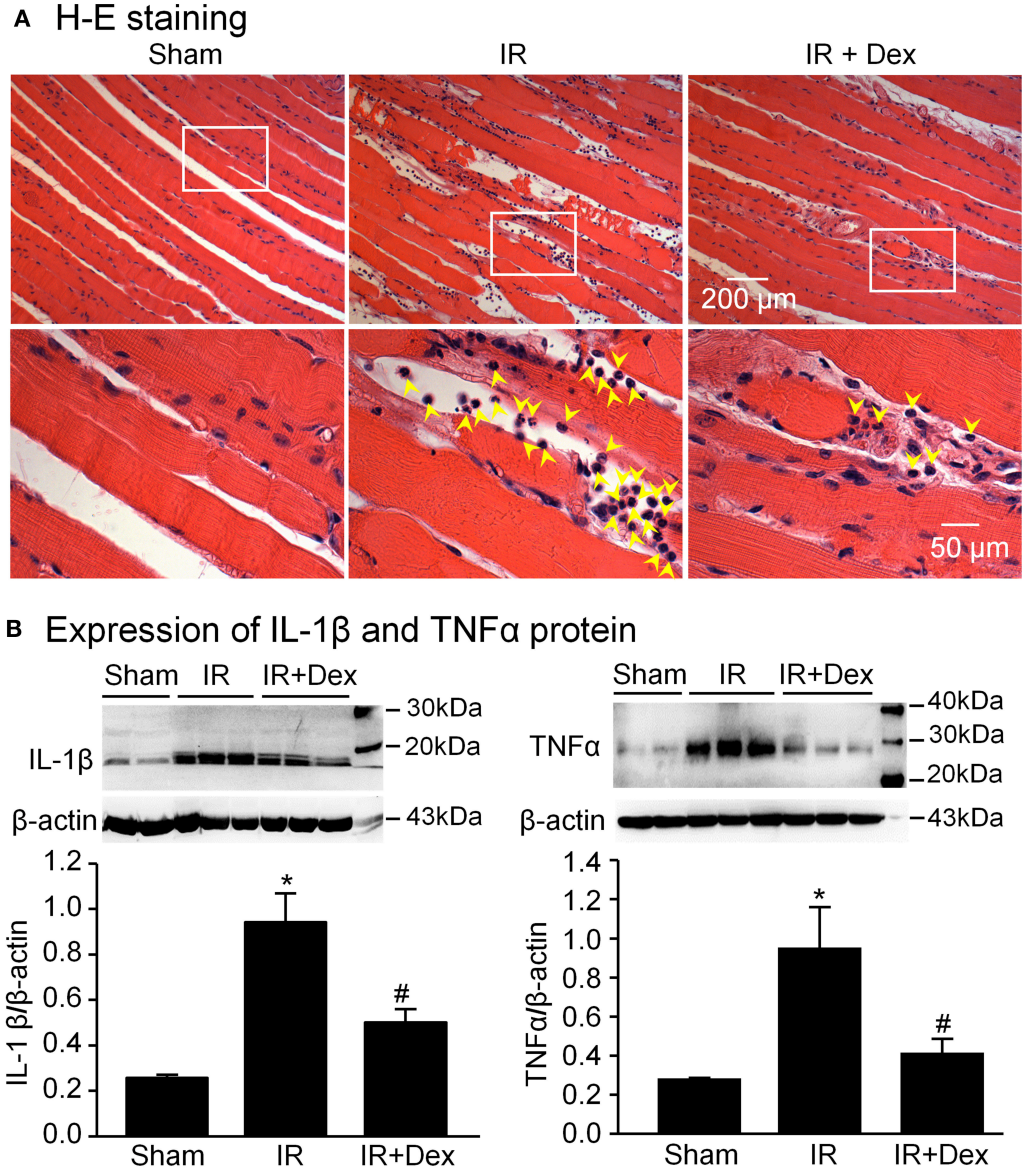
Leukocyte infiltration and expression of inflammatory cytokines in gastrocnemius muscles from all experimental groups. **(A)**, leukocyte infiltration into gastrocnemius muscles, measured by hematoxylin and eosin staining. Yellow arrowheads indicate leukocytes in gastrocnemius muscles. **(B)**, representative (upper panel) and summary (lower panel) data for expression of IL-1β and TNFα proteins in gastrocnemius muscles, measured by Western blot analysis. Data are mean ± SEM, *n* = 5 mice in each group. ^*^P < 0.05 vs. sham; ^#^*P* < 0.05 vs. IR.

In the sham group, there was no obvious leukocyte infiltration and expression of IL-1β and TNFα (2 proinflammatory cytokines) kept at a low level in the gastrocnemius muscle (Figure 5). However, after 3 h of tourniquet and 24 h of reperfusion, a large number of leukocytes, including neutrophils, lymphocytes, and monocytes were infiltrated into the IR-injured gastrocnemius muscle. Likewise, expression of IL-1β and TNFα significantly increased in the IR-injured gastrocnemius muscle. When Dex (1 mg/kg) was administered at the beginning of reperfusion, IR-induced leukocyte infiltration and overexpression of IL-1β and TNFα were markedly decreased (*p* < 0.05 vs. IR group, Figure 5).

## Discussion

In the present study, we mimicked the clinical tourniquet use to reproduce the critical limb syndrome in a mouse model subjected to 3 h of tourniquet and 24 h of reperfusion. In this mouse model, tourniquet-induced IR caused not only the morphological and functional damage of skeletal muscles and NMJs, but also the leukocyte infiltration and over-production of inflammatory cytokines in skeletal muscles. Treatment of Dex at the beginning of reperfusion significantly inhibited IR-induced leukocyte infiltration and overexpression of IL-1β and TNFα in gastrocnemius muscles. This anti-inflammatory drug also blunted acute IR-induced gastrocnemius muscle injuries and ameliorated direct muscle stimulation-evoked muscle contraction in IR-injured gastrocnemius muscles. However, Dex did not avert IR-induced disappearance of motor nerve terminals in NMJs from gastrocnemius muscles, and did not improve sciatic nerve-stimulated EPPs and muscle contraction in IR-injured gastrocnemius muscles. These results suggest that treatment of Dex at the beginning of reperfusion, through inhibiting inflammatory cytokines, alleviate acute IR-induced structural and functional impairments of gastrocnemius muscles but not NMJs.

There is a long history for tourniquet use in first-line treatment of severe limb hemorrhage. Although limited clinical evidence exists about complications associated with the use of this device due to the impossibility of distinguishing between tourniquet-induced and original hemorrhage-induced complications, concerns about tourniquet-induced nerve palsy, skeletal muscle injury, and amputation have led to the limitation of tourniquet use, suggesting as a last option after other hemorrhage control interventions fail (Clasper et al., 2009; Kue et al., 2015). Therefore, finding effective therapy for tourniquet-induced IR injuries can resolve the limitations of tourniquet use, and significantly improve outcomes and quality of life for this large group of patients.

Glucocorticoids such as Dex are widely used in clinical patients as potent anti-inflammatory drugs (Urbanska et al., 2014; Rodriguez et al., 2017). A previous study reported that the intramuscular administration of Dex 3 h prior to tourniquet successfully attenuated IR-induced muscle contractile dysfunction seen in rat extensor digitalis longus muscles following tourniquet-induced 3 h of ischemia and 24 h of reperfusion (Chen et al., 1996). As everyone knows, however, it is unrealistic to expect clinical pretreatment of drugs for tourniquet-induced IR injuries because extremity hemorrhage and tourniquet use are not predicted. To determine the clinical relevance of drugs, therefore, treatment of drugs at the beginning of reperfusion is an optimal timing for investigation of drugs' effects on tourniquet-induced IR injuries. In the present study, treatment of Dex at the beginning of reperfusion attenuated acute IR-induced gastrocnemius muscle injuries and improved direct muscle stimulation-evoked muscle contraction in IR-injured gastrocnemius muscles. It has been shown that Dex binding to the glucocorticoid receptor inhibits inflammatory cytokines through inhibition of NF-κB in some inflammatory conditions (Uddin et al., 2011). Our current study found that Dex significantly inhibited IR-induced overexpression of IL-1β and TNFα in gastrocnemius muscles. Analyzing above results, we thought that treatment of Dex at the beginning of reperfusion could improve the structure and function of skeletal muscles experienced 3 h of tourniquet and 24 h of reperfusion, through inhibition of inflammatory cytokine production. Nevertheless, treatment of Dex did not affect the structure and function of NMJs in IR-injured gastrocnemius muscles. It is possible that other cellular and molecular mechanisms (such as overproduction of reactive oxygen species and dysfunction of the endogenous antioxidative system) are responsible for acute IR-induced morphological and functional impairments of NMJs in the skeletal muscle, because G93A mutation of superoxide dismutase significantly increases the denervation of motor nerve terminals during skeletal muscle tourniquet-induced IR (David et al., 2007).

Inflammatory cytokines are mainly produced by activated leukocytes, including neutrophils, monocytes, and lymphocytes (Arango and Descoteaux, 2014). In the current study, tourniquet-induced IR caused the leukocyte infiltration into IR-injured gastrocnemius muscles. Treatment of Dex obviously blunted IR-induced leukocyte infiltration, which suggests that Dex decreased expression of IL-1β and TNFα in IR-injured gastrocnemius muscles, through inhibiting leukocyte activation and infiltration. It is possible that the skeletal muscle itself is also involved in IR-induced overexpression of IL-1β and TNFα, because the skeletal muscle is thought to be an endocrine organ for muscle-derived cytokine production (Pedersen and Febbraio, 2008; Borge et al., 2009; Arango and Descoteaux, 2014).

Our current study did not identify the contribution of each cytokine (IL-1β and TNFα) to tourniquet-induced IR injuries. Additionally, clinical use of Dex is hampered by its severe systemic side effects (Curtis et al., 2006; Fardet et al., 2007; Hanaoka et al., 2012). Therefore, the effects of IL-1 receptor antagonist (IL-1RA) and TNFα inhibitors (etanercept, infliximab, adalimumab, etc.) on tourniquet-induced IR injuries will be investigated in future study to discover the optimum clinical therapeutics.

In conclusion, tourniquet-induced IR results in morphological and functional impairments in mouse skeletal muscles and NMJs. Treatment of Dex at the beginning of reperfusion, through inhibiting inflammatory cytokines, protects the skeletal muscle from acute IR injuries, whereas it does not ameliorate the structure and function of NMJs in IR-injured skeletal muscles.

## Author contributions

RC, HT, DZ, and Y-LL designed and performed experiments, analyzed data, and prepared the manuscript. RC, AB, RM, MW, and Y-LL wrote, edited, and revised the manuscript. All authors approved the version to be published.

### Conflict of interest statement

The authors declare that the research was conducted in the absence of any commercial or financial relationships that could be construed as a potential conflict of interest.

## References

[B1] Aho K. Sainio K. Kianta M. Varpanen E. (1983). Pneumatic tourniquet paralysis. case report. J. Bone Joint Surg. Br. 65, 441–443. 10.1302/0301-620X.65B4.6874716 6874716

[B2] Arango D. G. Descoteaux A. (2014). Macrophage cytokines: involvement in immunity and infectious diseases. Front. Immunol. 5:491 10.3389/fimmu.2014.00491 25339958 PMC4188125

[B3] Barnes P. J. (2011). Glucocorticosteroids: current and future directions. Br. J. Pharmacol. 163, 29–43. 10.1111/j.1476-5381.2010.01199.x 21198556 PMC3085866

[B4] Beekley A. C. Sebesta J. A. Blackbourne L. H. Herbert G. S. Kauvar D. S. Baer D. G.. (2008). Prehospital tourniquet use in operation Iraqi freedom: effect on hemorrhage control and outcomes. J. Trauma 64, S28–S37. 10.1097/TA.0b013e318160937e 18376169

[B5] Borge B. A. Kalland K. H. Olsen S. Bletsa A. Berggreen E. Wiig H. (2009). Cytokines are produced locally by myocytes in rat skeletal muscle during endotoxemia. Am. J. Physiol. Heart Circ. Physiol. 296, H735–H744. 10.1152/ajpheart.01309.2008 19151248

[B6] Center for Disease Control Prevention (2017). Estimated Number of Extremities Visited to Ed and Cost of Injury Reports. Available online at: https://wisqars.cdc.gov:8443/costT/

[B7] Chen G. Y. Nunez G. (2010). Sterile inflammation: sensing and reacting to damage. Nat. Rev. Immunol. 10, 826–837. 10.1038/nri2873 21088683 PMC3114424

[B8] Chen L. E. Silver W. P. Seaber A. V. Korompilias A. V. Urbaniak J. R. (1996). Effects of dexamethasone on the contractile function of reperfused skeletal muscle. Microsurgery 17, 313–320. 10.1002/(SICI)1098-2752(1996)17:6<313::AID-MICR5>3.0.CO;2-I 9308715

[B9] Chervu A. Moore W. S. Homsher E. Quinones-Baldrich W. J. (1989). Differential recovery of skeletal muscle and peripheral nerve function after ischemia and reperfusion. J. Surg. Res. 47, 12–19. 10.1016/0022-4804(89)90041-3 2739397

[B10] Clasper J. C. Brown K. V. Hill P. (2009). Limb complications following pre-hospital tourniquet use. J. R. Army Med. Corps 155, 200–202. 10.1136/jramc-155-03-06 20397360

[B11] Clementsen T. Reikeras O. (2008). Cytokine patterns after tourniquet-induced skeletal muscle ischaemia reperfusion in total knee replacement. Scand. J. Clin. Lab. Invest. 68, 154–159. 10.1080/00365510701528587 17963155

[B12] Curtis J. R. Westfall A. O. Allison J. Bijlsma J. W. Freeman A. George V.. (2006). Population-based assessment of adverse events associated with long-term glucocorticoid use. Arthritis Rheum. 55, 420–426. 10.1002/art.21984 16739208

[B13] David G. Nguyen K. Barrett E. F. (2007). Early vulnerability to ischemia/reperfusion injury in motor terminals innervating fast muscles of SOD1-G93A mice. Exp. Neurol. 204, 411–420. 10.1016/j.expneurol.2006.12.021 17292357 PMC2097955

[B14] Eastlack R. K. Groppo E. R. Hargens A. R. Pedowitz R. A. (2004). Ischemic-preconditioning does not prevent neuromuscular dysfunction after ischemia-reperfusion injury. J. Orthop. Res. 22, 918–923. 10.1016/j.orthres.2003.10.015 15183455

[B15] Eltzschig H. K. Eckle T. (2011). Ischemia and reperfusion–from mechanism to translation. Nat. Med. 17, 1391–1401. 10.1038/nm.2507 22064429 PMC3886192

[B16] Fardet L. Kassar A. Cabane J. Flahault A. (2007). Corticosteroid-induced adverse events in adults: frequency, screening and prevention. Drug Saf. 30, 861–881. 10.2165/00002018-200730100-00005 17867724

[B17] Fish J. S. McKee N. H. Pynn B. R. Kuzon W. M. Jr. Plyley M. J. (1989). Isometric contractile function recovery following tourniquet ischemia. J. Surg. Res. 47, 365–370. 10.1016/0022-4804(89)90149-2 2770293

[B18] Gillani S. Cao J. Suzuki T. Hak D. J. (2012). The effect of ischemia reperfusion injury on skeletal muscle. Injury 43, 670–675. 10.1016/j.injury.2011.03.008 21481870

[B19] Halladin N. L. Zahle F. V. Rosenberg J. Gogenur I. (2014). Interventions to reduce tourniquet-related ischaemic damage in orthopaedic surgery: a qualitative systematic review of randomised trials. Anaesthesia 69, 1033–1050. 10.1111/anae.12664 24800642

[B20] Hanaoka B. Y. Peterson C. A. Horbinski C. Crofford L. J. (2012). Implications of glucocorticoid therapy in idiopathic inflammatory myopathies. Nat. Rev. Rheumatol. 8, 448–457. 10.1038/nrrheum.2012.85 22688888

[B21] Inaba K. Siboni S. Resnick S. Zhu J. Wong M. D. Haltmeier T.. (2015). Tourniquet use for civilian extremity trauma. J. Trauma Acute Care Surg. 79, 232–237. 10.1097/TA.0000000000000747 26218691

[B22] Kam P. C. Kavanagh R. Yoong F. F. (2001). The arterial tourniquet: pathophysiological consequences and anaesthetic implications. Anaesthesia 56, 534–545. 10.1046/j.1365-2044.2001.01982.x 11412159

[B23] Kragh J. F. Jr. Swan K. G. Smith D. C. Mabry R. L. Blackbourne L. H. (2012). Historical review of emergency tourniquet use to stop bleeding. Am. J. Surg. 203, 242–252. 10.1016/j.amjsurg.2011.01.028 21782152

[B24] Kue R. C. Temin E. S. Weiner S. G. Gates J. Coleman M. H. Fisher J.. (2015). Tourniquet use in a civilian emergency medical services setting: a descriptive analysis of the boston EMS experience. Prehosp. Emerg. Care 19, 399–404. 10.3109/10903127.2014.995842 25665102

[B25] Lotfi S. Patel A. S. Mattock K. Egginton S. Smith A. Modarai B. (2013). Towards a more relevant hind limb model of muscle ischaemia. Atherosclerosis 227, 1–8. 10.1016/j.atherosclerosis.2012.10.060 23177969

[B26] Mabry R. L. (2006). Tourniquet use on the battlefield. Mil. Med. 171, 352–356. 10.7205/MILMED.171.5.352 16761880

[B27] Mohler L. R. Pedowitz R. A. Lopez M. A. Gershuni D. H. (1999). Effects of tourniquet compression on neuromuscular function. Clin. Orthop. Relat. Res. 213–220. 10.1097/00003086-199902000-00024 10078146

[B28] Noordin S. McEwen J. A. Kragh J. F. Jr. Eisen A. Masri B. A. (2009). Surgical tourniquets in orthopaedics. J. Bone Joint Surg. Am. 91, 2958–2967. 10.2106/JBJS.I.00634 19952261

[B29] Ode G. Studnek J. Seymour R. Bosse M. J. Hsu J. R (2015). Emergency tourniquets for civilians: can military lessons in extremity hemorrhage be translated?. J. Trauma Acute Care Surg. 79, 586-591. 10.1097/TA.0000000000000815 26402532

[B30] Pedersen B. K. Febbraio M. A. (2008). Muscle as an endocrine organ: focus on muscle-derived interleukin-6. Physiol. Rev. 88, 1379–1406. 10.1152/physrev.90100.2007 18923185

[B31] Rodriguez V. J. Rodriguez V. L. Guzman N. M. (2017). Pharmaceutical technology can turn a traditional drug, dexamethasone into a first-line ocular medicine. a global perspective and future trends. Int. J. Pharm. 516, 342–351. 10.1016/j.ijpharm.2016.11.053 27889587

[B32] Schoen M. Rotter R. Gierer P. Gradl G. Strauss U. Jonas L. (2007). Ischemic preconditioning prevents skeletal muscle tissue injury, but not nerve lesion upon tourniquet-induced ischemia. J. Trauma 63, 788–797. 10.1097/01.ta.0000240440.85673.fc 18090007

[B33] Schuld A. Kraus T. Haack M. Hinze-Selch D. Zobel A. W. Holsboer F.. (2001). Effects of dexamethasone on cytokine plasma levels and white blood cell counts in depressed patients. Psychoneuroendocrinology 26, 65–76. 10.1016/S0306-4530(00)00039-1 11070335

[B34] Schwiebert L. M. Beck L. A. Stellato C. Bickel C. A. Bochner B. S. Schleimer R. P. (1996). Glucocorticosteroid inhibition of cytokine production: relevance to antiallergic actions. J. Allergy Clin. Immunol. 97, 143–152. 10.1016/S0091-6749(96)80214-4 8568145

[B35] Seekamp A. Warren J. S. Remick D. G. Till G. O. Ward P. A. (1993). Requirements for tumor necrosis factor-alpha and interleukin-1 in limb ischemia/reperfusion injury and associated lung injury. Am. J. Pathol. 143, 453–463. 7688184 PMC1887029

[B36] Smits H. H. Grunberg K. Derijk R. H. Sterk P. J. Hiemstra P. S. (1998). Cytokine release and its modulation by dexamethasone in whole blood following exercise. Clin. Exp. Immunol. 111, 463–468. 10.1046/j.1365-2249.1998.00482.x 9486420 PMC1904898

[B37] Tai T. W. Lin C. J. Jou I. M. Chang C. W. Lai K. A. Yang C. Y. (2011). Tourniquet use in total knee arthroplasty: a meta-analysis. Knee Surg. Sports Traumatol. Arthrosc. 19, 1121–1130. 10.1007/s00167-010-1342-7 21161177 PMC3116117

[B38] Tran T. P. Tu H. Liu J. Muelleman R. L. Li Y. L. (2012). Mitochondria-derived superoxide links to tourniquet-induced apoptosis in mouse skeletal muscle. PLoS ONE 7:e43410. 10.1371/journal.pone.0043410 22912870 PMC3422247

[B39] Tran T. P. Tu H. Pipinos I. I. Muelleman R. L. Albadawi H. Li Y. L. (2011). Tourniquet-induced acute ischemia-reperfusion injury in mouse skeletal muscles: Involvement of superoxide. Eur. J. Pharmacol. 650, 328–334. 10.1016/j.ejphar.2010.10.037 21036124 PMC3008320

[B40] Tu H. Zhang D. Corrick R. M. Muelleman R. L. Wadman M. C. Li Y. L. (2017). Morphological regeneration and functional recovery of neuromuscular junctions after tourniquet-induced injuries in mouse hindlimb. Front. Physiol. 8:207. 10.3389/fphys.2017.00207 28428759 PMC5382216

[B41] Uddin M. N. Siddiq A. Oettinger C. W. D'Souza M. J. (2011). Potentiation of pro-inflammatory cytokine suppression and survival by microencapsulated dexamethasone in the treatment of experimental sepsis. J. Drug Target 19, 752–760. 10.3109/1061186X.2011.561856 21913870

[B42] Urbanska J. Karewicz A. Nowakowska M. (2014). Polymeric delivery systems for dexamethasone. Life Sci. 96, 1–6. 10.1016/j.lfs.2013.12.020 24373835

[B43] Vignaud A. Hourde C. Medja F. Agbulut O. Butler-Browne G. Ferry A. (2010). Impaired skeletal muscle repair after ischemia-reperfusion injury in mice. J. Biomed. Biotechnol. 2010:724914. 10.1155/2010/724914 20467471 PMC2866363

[B44] Walters T. J. Kragh J. F. Baer D. G. (2008). Influence of fiber-type composition on recovery from tourniquet-induced skeletal muscle ischemia-reperfusion injury. Appl. Physiol. Nutr. Metab. 33, 272–281. 10.1139/H07-180 18347682

[B45] Welling D. R. McKay P. L. Rasmussen T. E. Rich N. M. (2012). A brief history of the tourniquet. J. Vasc. Surg. 55, 286–290. 10.1016/j.jvs.2011.10.085 22183005

[B46] Woitaske M. D. McCarter R. J. (1998). Effects of fiber type on ischemia-reperfusion injury in mouse skeletal muscle. Plast. Reconstr. Surg. 102, 2052–2063. 10.1097/00006534-199811000-00037 9811003

[B47] Zhang D. Wang D. Pipinos I. I. Muelleman R. L. Li Y. L. (2017). Dexamethasone promotes long-term functional recovery of neuromuscular junction in a murine model of tourniquet-induced ischaemia-reperfusion. Acta Physiol. 219, 453–464. 10.1111/apha.12737 27306588

